# Trauma-informed prevention programmes for depression, anxiety, and substance use among young people: protocol for a mixed-methods systematic review

**DOI:** 10.1186/s13643-023-02365-4

**Published:** 2023-10-31

**Authors:** S. Bailey, N. Newton, Y. Perry, L. Grummitt, L. Baams, E. Barrett

**Affiliations:** 1 The Matilda Centre for Research in Mental Health and Substance Use, Faculty of Medicine and Health, University of Sydney, Sydney, Australia; 2 Telethon Kids Institute, University of Western Australia, Perth, Australia; 3 University of Groningen, Groningen, Netherlands

**Keywords:** Mental health, Substance use, Trauma-informed, Young people, Prevention

## Abstract

**Background:**

Mental ill-health and substance use bear a substantial burden and harm on young people and often arise from co-occurring and compounding risk factors, such as traumatic stress. Trauma-informed prevention of mental ill-health and substance use demonstrates significant promise in reducing this burden. A systematic literature review is required to identify and summarise the effectiveness, feasibility, acceptability, and design principles underpinning existing trauma-informed mental ill-health and/or substance use prevention programmes for young people aged 12–24 years.

**Methods:**

MEDLINE, Embase, CINAHL, PsychINFO, and Cochrane Library will be searched from 2012 through September 2022. Reference lists of included articles will be citation-chained. Title and abstracts will be screened and two reviewers will review articles full-text. One reviewer will extract data from eligible articles using a piloted data extraction form, and 20% of the data will be verified by a second reviewer. Risk of bias will be assessed using the Cochrane risk-of-bias tool for randomised trials (RoB 2), Risk of Bias in Non-randomised Studies of Interventions (ROBINS-I), and The Joanna Briggs Institute Critical Appraisal Checklist for Quasi-Experimental Studies and The Joanna Briggs Institute Critical Appraisal Checklist for Qualitative Research (CASP), depending on the study type. Characteristics of existing trauma-informed mental ill-health and/or substance use prevention programmes for young people will be summarised narratively. Effectiveness, feasibility, and acceptability will be qualitatively described and summarised, with proportions and effect sizes quantitatively synthesised, where possible.

**Discussion:**

Trauma-informed approaches to prevention demonstrate significant promise, yet to date, no study has systematically summarised and synthesised the available literature. To fill this gap, the present review will systematically identify and summarise the effectiveness, feasibility, acceptability, and design principles underpinning existing trauma-informed mental health and/or substance use prevention programmes for young people aged 12–24. This review will inform the development, adaptation, evaluation, and implementation of future trauma-informed mental ill-health and substance use prevention programmes for young people. Findings will inform critical efforts to interrupt and prevent already elevated trajectories of mental ill-health, substance use, and related harms among those young people exposed to adversity.

**Systematic review registration:**

PROSPERO CRD42022353883.

## Introduction

Mental ill-health and substance use are among the leading causes of injury, morbidity, disability, and mortality among young people globally [1, 2]. Mental ill-health and substance use often co-occur among young people and compound adverse health, social, and economic effects at individual, family, and community levels [3–5]. Mental ill-health and substance use during developmental years are prospectively associated with the development, onset, and prognosis of mental health and substance use disorders in adulthood [3–6]. In fact, over 60% of these disorders develop before early adulthood [7–9]. Further prevention research is required to address co-occurring mental ill-health and substance use among young people and prevent their associated harms.

Accumulating, robust evidence supports the effectiveness, feasibility, acceptability, and scalability of mental ill-health and substance use prevention programmes for young people [10, 11]. Specifically, the literature highlights the potential of two distinct approaches to prevention: ‘universal’ programmes (open to all irrespective of risk levels) [12–14] and ‘selective’ programmes (most at-risk targeted) [15]. Recent analyses indicate these universal and selective programmes can confer long-term benefits, preventing mental ill-health symptoms and substance misuse through early adulthood [16, 17]. These universal and selective prevention programmes are commonly school-based and delivered online, combining prevention messaging with valuable skills training to prevent and reduce co-occurring mental ill-health and substance use [18, 19]. Notwithstanding this, the key to the expansion and scaling of these prevention programmes is the identification and targeting of emerging risk and protective factors [11].

Evidence accumulated over the last few decades has identified one such risk factor, traumatic stress, which is highly prevalent and strongly associated with mental ill-health and substance use in adolescence and early adulthood [20–24]. For the purpose of this review, trauma includes exposure to violence or actual or threatened death [25] and other adverse experiences, such as emotional abuse and neglect [26]. A recent review of international evidence indicates almost two-thirds of school-aged young people experience adverse events [27]. For the purposes of this review, exposure to traumatic events is of interest rather than the potential impacts of the traumatic events. Trauma is positively associated with the development and onset of mental health and substance use disorders [22, 28, 29]. Addressing trauma among young people is critical for effective mental ill-health and substance use prevention efforts.

Trauma-informed approaches for preventing mental ill-health and substance use demonstrate significant promise [30]. Trauma-informed prevention realises, recognises, and responds to trauma, its signs, symptoms, and impacts, actively resisting re-traumatisation [31, 32]. In doing so, trauma-informed approaches cultivate resilience and empower individuals to mitigate the impact of their adverse experiences [32]. For example, the trauma-informed electronic cigarette prevention programme, ‘Rise Above’, aims to provide youth with social and emotional skills in conjunction with substance use knowledge and resistance skills [33]. While this definition of trauma-informed is generated from the treatment field, this definition is increasingly being applied to prevention due to its wide applicability and guiding principles. Unlike traditional public health prevention, experts recommend that trauma-informed prevention programmes provide psychoeducation regarding trauma and its impacts and relevant skill-building to develop healthy strategies to cope and enhance social and emotional competencies [33–36]. Notwithstanding this, however, much of the literature to date focuses largely on early intervention and treatment as opposed to prevention [23, 37]. Available trauma-informed prevention literature is sparse and tends to focus on tobacco, e-cigarettes, and suicide prevention [30, 33–35]. Limited studies focus on groups of individuals more at risk of experiencing trauma, including gender and sexuality-diverse (LGBTQ +) and First Nations young people. Inclusive and tailored trauma-informed prevention interventions are especially important for these individuals due to their experiencing disproportionately higher levels of mental ill-health and/or substance use [38, 39]. Conversely, considerable work examines the utility of trauma-informed school environments for mitigating the effects of childhood adversity on educational outcomes; however, the preventative effects of these programmes do not specifically target mental ill-health symptoms or substance use behaviours [40].

Given the clear need for trauma-informed mental ill-health and/or substance use prevention, the present review aims to identify and systematically describe existing trauma-informed mental ill-health and/or substance use prevention programmes for young people through a systematic review of the peer-reviewed and grey literature. This review is concerned with the design and evaluation of existing trauma-informed mental ill-health and/or substance use prevention programmes for young people. Two research questions guide this review: (1) what is the effectiveness, feasibility, or acceptability of existing trauma-informed mental ill-health and/or substance use prevention programmes for young people? and (2) what are the trauma-informed design characteristics of these programmes?

## Methods

### Eligibility criteria

#### Study characteristics

##### Participants

Eligible participants include young people aged 12–24 years from setting or geographical location in the world.

##### Intervention

This review is concerned with the design and evaluation of existing trauma-informed substance use and/or mental ill-health prevention programmes.

Prevention programmes at universal (delivered to all), selective (delivered to those at increased risk), and indicated (delivered to those with elevated symptoms) are eligible for inclusion. Tertiary-level prevention programmes (those which aim to reduce symptoms after clinical diagnosis) will be excluded from the review. Studies detailing trauma-informed mental health treatment or service provision are outside the scope of the present review and will be excluded. Studies detailing treatment or service provision for substance use among people with exposure to traumatic experiences will also be excluded. Studies that detail trauma prevention programmes (e.g. violence prevention) with secondary mental ill-health and/or substance use outcomes will only be included if they meet the aforementioned definition of being trauma-informed. The main point of delineation between treatment programmes versus the prevention programmes of interest to this review is that prevention programmes aim to prevent the onset of depression, anxiety, and substance use and/or early detection and intervention before symptoms or substance use is present, whereas treatment programmes aim to treat clinically significant levels of depressive/anxiety symptoms and/or harmful substance use [19].

Definitions of ‘trauma-informed’ approaches vary significantly [41]. To identify trauma-informed approaches, this review requires eligible articles describing programmes that meet one of three criteria: (i) programme demonstrates knowledge and understanding of the impact of trauma on participants, (ii) programme contains skill-based component to foster social and emotional strength, and (iii) programme contains a psychoeducational component regarding the relationship between trauma and mental ill-health and/or substance use. These criteria are based on findings from recent trauma-informed methodological studies [33, 42, 43] as well as seminal technical handbooks regarding trauma-informed approaches, including the Substance Abuse and Mental Health Services Administration (SAMHSA) [31, 32, 44]. Our three-pronged criteria align directly with three of the four principles outlined in SAMHSA’s trauma-informed approach guideline: demonstrating knowledge and understanding of trauma impacts (‘Realisation and Recognition’), providing skill-based component to foster social and emotional strength (‘Responding’), and incorporating a psychoeducational component regarding the relationship between trauma and mental ill-health/substance use (‘Recognition’), respectively. While the present review operationalises trauma-informed to align with three of the four tenets of trauma-informed approaches as defined by the SAMHSA, it is important to note that the fourth tenet relating to Resist Retraumatisation (‘actively resisting re-traumatisation’) is a necessary condition of programmes meeting criterion (i) related to providing knowledge and understanding of the impacts of trauma. For example, through demonstrating the signs, symptoms, and impacts of cisnormative and heterosexual minority stress on LGBTQ + young people, including incidents of bullying and discrimination, programmes such as AFFIRM Online [45] and Proud and Empowered [46] actively resist re-traumatising the LGBTQ + programme participants.

##### Comparator

Appropriate comparators of interest to this review include age-matched (12–24 years) young people who do not participate in eligible trauma-informed prevention programmes or receive control condition alternatives, for example, health education as usual.

##### Outcome

The present review limits the scope of mental ill-health prevention programmes to symptoms and feelings of depression and anxiety. These symptoms and associated disorders represent the largest burden of mental ill-health among young people [2].

The present review will include prevention programmes targeting licit and illicit drugs as a substantial proportion of the burden of disease related to substance use among young people is attributable to alcohol, tobacco, cannabis, and common illicit drugs such as ecstasy and cocaine.

Accordingly, we designed our search terms to capture preventative programmes targeting any anxiety symptoms/disorders, any depressive symptoms/disorders, and any substance use. While depressive symptoms may rise to the level of mood disorders, these three mental ill-health conditions were purposely chosen as these account for a majority of the burden of disease attributable to trauma among young people globally [47], are the three most prevalent mental health disorders in this age group [48], and are strongly associated outcomes of exposure to trauma [26]. Given the main outcomes of interest related to anxiety and depression and substance use more broadly, prevention programmes explicitly targeting post-traumatic stress disorder symptoms were not the focus of this review. Future research could consider other programmes designed to prevent other psychological sequelae associated with trauma such as post-traumatic stress disorder, complex trauma, and borderline personality disorder.

All measures including qualitative descriptions, non-standardised measures, and standardised measures, of depressive and anxiety symptoms are eligible for the evaluation of the effectiveness, acceptability, and feasibility of programmes within included studies. All measures must be sourced from the young person participating in the prevention programme, i.e. self-reported. Exposure-based measures of substance use, including ever use, recent use, and frequency, are all acceptable for the purposes of this review.

Studies must report at least one of the outcomes of interest to be eligible.

##### Study designs

Randomised controlled trials, non-randomised controlled trials, pre-post designs, and qualitative studies are all eligible study designs for the purposes of determining the effectiveness of included interventions. Qualitative studies will be deemed acceptable for the purposes of examining the acceptability and feasibility of trauma-informed prevention programmes within the included studies.

#### Report characteristics

Articles published from 2012 through September 2022 will be searched. This date range has been selected to reflect the relative recency of trauma-informed research [49]. Articles available in full-text will be considered. Conference abstracts will therefore be excluded. Commentary, opinion-style, and review articles will be excluded.

##### Information sources

We will search for relevant peer-reviewed literature in the Cochrane Library, PsychINFO, CINAHL, Embase, and MEDLINE database reference platforms. There are no language restrictions for the present review. Citation chaining of reference lists of included articles will ensure relevant literature is identified. Through the authors’ professional networks, expert researchers and clinicians within the field of trauma-informed prevention will be contacted to source additional papers not identified within the proposed database search strategy.

##### Search strategy

A medical specialist librarian from Fisher Library at The University of Sydney will be iteratively consulted to develop our search strategy. Table 1 displays the proposed search strategy for peer-reviewed literature, comprising search terms within the five main constructs underpinning the review research questions: (i) trauma-informed, (ii) mental ill-health, (iii) substance use, (iv) prevention, and (v) young people. In conjunction with general search terms, database-specific search terms and related Boolean operators will be used. Grey literature searching will use iterative combinations of the below terms (Table 1). For illustrative purposes, an example search strategy run in the MEDLINE database reads as follows: (trauma-informed OR trauma-sensitive OR trauma-aware* OR posttrauma* OR adverse childhood experience* OR child maltreatment “Stress disorders, Post-Traumatic” OR “Child Abuse” OR “Psychological Trauma” OR “Adverse Childhood Experiences”) AND (mental health OR depressi* OR anxi* OR Mental Health OR Depression OR Anxiety OR Anxiety Disorders OR substance-use* OR alcohol misuse* OR cannabis* OR tobacco* OR vaping* OR electronic cigarette* OR e-cigarette* OR illicit drug* OR “methamphetamine*” OR ecstasy* OR opioid* OR alcohol* OR amphetamine OR cocaine* OR opiate* OR heroin* OR inhalant* OR marijuana OR LSD OR MDMA OR GHB OR ketamine OR kava OR steroid* OR NPS OR hallucinogen* OR benzodiazepine* OR stimulant* OR “Substance-Related Disorders”[MeSH]) AND (prevention OR education* OR program* OR campaign* OR curriculum* OR “Primary Prevention”[MeSH] OR “Secondary Prevention”[MeSH] OR ((health* OR prevention* OR education*) adj3 (program* OR design* OR implementat* OR evaluat* OR strateg* OR initiative* OR campaign* OR promot* OR curriculum* OR policy OR policies)) AND (youth OR adolescen* OR teen* OR “young people” OR child OR “young adult*” OR Adolescent OR Young Adult OR Child). No limits or filters will be applied to searches.

**Table 1:** Search strategy keywords, alternative terms, and database-specific search terms^a^

**Concept group**	**Search terms**	**Medical Subject Headings (MeSH) terms (PubMed)**^a^	**American Psychological Association Index Terms (PsychINFO)**	**Emtree terms (Embase)**	**CINAHL Subject Headings (CINAHL Complete)**
1. Trauma-informed	trauma-informed OR trauma-sensitive OR trauma-aware^a^ OR posttrauma^a^ OR adverse childhood experience^a^ OR child maltreatment	‘Stress disorders, Post-Traumatic’ OR ‘Child Abuse’ OR ‘Psychological Trauma’ OR ‘Adverse Childhood Experiences’	‘Trauma’ OR ‘Trauma-Informed Care’ OR ‘Emotional Trauma’ OR ‘child Abuse’ OR ‘Childhood Adversity’	‘Posttraumatic Stress Disorder’ OR ‘Childhood Adversity’ OR ‘Psychotrauma’ OR ‘Child Abuse’	‘Adverse Childhood Experiences’ OR ‘Psychological Trauma’ OR ‘Child Abuse’
2. Mental ill-health	mental health OR depressi^a^ OR anxi^a^	Mental Health OR Depression OR Anxiety OR Anxiety Disorders	Depression (Emotion) OR Anxiety OR Anxiety Disorders	Depression OR Anxiety OR Anxiety Disorder	Depression OR Anxiety OR Anxiety Disorders
3. “Substance use^a^”	substance-use^a^ OR alcohol misuse^a^ OR cannabis^a^ OR tobacco^a^ OR vaping^a^ OR electronic cigarette^a^ OR e-cigarette^a^ OR illicit drug^a^ OR “methamphetamine^a^” OR ecstasy^a^ OR opioid^a^ OR alcohol^a^ OR amphetamine OR cocaine^a^ OR opiate^a^ OR heroin^a^ OR inhalant^a^ OR marijuana OR LSD OR MDMA OR GHB OR ketamine OR kava OR steroid^a^ OR NPS OR hallucinogen^a^ OR benzodiazepine^a^ OR stimulant^a^	‘Substance-Related Disorders’[MeSH]	‘Substance Use Disorder’ OR ‘Substance Use Prevention’	‘Substance Use’ OR ‘Substance Abuse’	‘Substance Use Disorders’ OR ‘Substance USE’
4. Prevention	prevention OR education^a^ OR program^a^ OR campaign^a^ OR curriculum^a^	‘Primary Prevention’[MeSH] OR ‘Secondary Prevention’[MeSH] OR ((health^a^ OR prevention^a^ OR education^a^) adj3 (program^a^ OR design^a^ OR implementat^a^ OR evaluat^a^ OR strateg^a^ OR initiative^a^ OR campaign^a^ OR promot^a^ OR curriculum^a^ OR policy OR policies))	‘Harm Reduction’ OR ‘Prevention’ OR ‘Relapse Prevention’ OR ((health^a^ OR prevention^a^ OR education^a^) adj3 (program^a^ OR design^a^ OR implementation^a^ OR evaluation^a^ OR strateg^a^ OR initiative^a^ OR campaign^a^ OR promot^a^ OR curriculum^a^))	‘Prevention’ OR ‘Prevention Study’ OR ‘Primary Prevention’ OR ‘Secondary Prevention’ OR ‘Smoking Prevention’ OR ((health^a^ OR prevention^a^ OR education^a^) adj3 (program^a^ OR design^a^ OR implementation^a^ OR evaluation^a^ OR strateg^a^ OR initiative^a^ OR campaign^a^ OR promot^a^ OR curriculum^a^))	‘Substance Use Prevention’
5. Young people	youth OR adolescen^a^ OR teen^a^ OR “young people” OR child OR “young adult^a^”	Adolescent OR Young Adult OR Child		Adolescent OR Child OR Young Adult	Young Adult OR Child

^a^The five domains detailed in Table 1 are connected via the use of Boolean operators in the following manner: (1 AND (2 OR 3) AND 4 AND 5)

### Study records

#### Data management

Identified articles will be downloaded to Covidence (systematic review management software; http://www.covidence.org/). Only project staff members will have access to this password-protected Covidence file.

#### Selection process

Article titles and abstracts returned by the databases in response to the search terms were imported into Covidence, which was used for title/abstract screening as well as full-text screening. All titles/abstracts will be screened by one reviewer. A second reviewer will screen 10% of titles and abstracts. Using Covidence functionality, inter-rater reliability for overlapping articles will be calculated. A ‘substantial’ threshold of Kappa’s Cohen 0.61–0.80 will be set. A second reviewer will continue screening a random selection of additional titles and abstracts until inter-rater reliability is above acceptable threshold. If the threshold is not met, the second reviewer will keep screening until the threshold is met. Where inter-rater reliability is not achieved, both reviewers will meet with a third reviewer to discuss and clarify study eligibility. In the event that an article is deemed potentially eligible or not enough information is provided in the title and abstract to determine eligibility, this article will be subject to full-text review. Two reviewers will review the full text of eligible articles. This full-text screening will focus on the PICOS elements outlined explicitly in the present protocol. Where screening decisions differ, both reviewers will discuss until consensus is achieved. Where consensus is not reached, a third reviewer will make the final screening/inclusion decision.

#### Data collection process

Following screening, included articles will undergo data extraction using Covidence in accordance with a standardised data extraction form informed by preliminary literature scans which will be finalised prior to executing the search strategies. This form will be piloted to ensure all details relevant to the research questions are captured. To minimise error in data extraction, one reviewer will data extract all included articles, and a second reviewer will randomly select and verify 20% of the total number of included data extraction records. Where there is missing data from included studies, the authors will attempt to contact study authors for further detail or clarification. Upon completion of data extraction using Covidence, results will be exported into Microsoft Excel to facilitate ease of data viewing and summarising.

#### Study characteristics of interest

Study characteristics for which data will be extracted are detailed in Table 2, including their definitions.

**Table 2:** List and definitions of study characteristics for which data will be extracted

Data item	Definition
Title	Title of publication
Publication date	Date of publication
Authors	Publication authors
Setting	Location of programme implementation, including geographical location
Sample characteristics—basic demographics	Standard demographic factors of study participants: age, sex, gender
Sample characteristics—marginalised group membership	Marginalised group identity, e.g. LGBTQA + , Aboriginal and/or Torres Strait Islander young people
Sample size	How many participants were included in the study?
Sample method	How were programme participants identified and selected?
Study design	Study design employed
Comparison	The comparison group used, if applicable
Research translation position	Where along the research translation spectrum the article sits: knowledge generation, intervention development, intervention effectiveness, intervention implementation, intervention evaluation

#### Outcomes of interest

Analysis of included articles will focus on factors related to the two research questions: (1) effectiveness, feasibility, and acceptability levels of existing trauma-informed mental health and/or substance use prevention programmes, and (2) the trauma-informed programme design characteristics.

Tables 3, 4, and 5 detail the intervention characteristics of interest.

**Table 3:** Intervention characteristics with definitions and rationales

Participants	Who were programme participants? E.g. child, parent/guardian	To identify participants targeted by available interventions
Eligibility requirements	Were there eligibility requirements for programme participation? If so, what were they?	To analyse the access and reach of available interventions
Programme name	What was the programme called? (If applicable)	To identify the names of available interventions
Programme components and delivery	What components comprise this programme and how are they delivered?	To identify components of a successful trauma-informed drug prevention programme
Programme aim	What was the stated aim of the programme?	To scope the aims of available programmes
Programme mode of delivery	How are the programme components delivered?	To identify optimal delivery of trauma-informed prevention programme
Prevention scope	What risk and protective factors for mental ill-health and/or substance use does this programme target?	To evaluate how trauma-informed prevention differs by target
Underpinning theory	What is the underpinning theory of the programme?	To scope the underpinning theories of trauma-informed prevention programmes
Follow-up	Were programme participants followed up? If so, how?	To evaluate short and long-term effects of interventions

**Table 4:** Trauma-informed intervention characteristics with definitions and rationales

Outcome	Definition	Rationale
Rationale	Do the authors state a rationale for this programme being *trauma-informed*?	To build a standardised recommendation and case for trauma-informed programme design
Trauma type	What types of traumatic exposures inform this programme?	To identify common and uncommon traumatic exposures accounted for in current trauma-informed prevention
Trauma-informed characteristics	What programme characteristics embody ‘trauma-informed’ principles?	To analyse what programme characteristics optimally reflect trauma-informed design
Trauma-informed adaptation and implementation	Was the programme designed trauma-informed or adapted to be trauma-informed? If so, how?	To evaluate best-practice adaptation and implementation of trauma-informed programme design
Trauma-informed design guideline	What guideline, if any, was used to design this trauma-informed programme?	To identify best-practice guideline where available
Trauma-informed training requirement	What training requirements, if any, were there for those involved with programme delivery?	To analyse training and education needs (met and unmet) for trauma-informed prevention programme personnel

**Table 5:** Intervention outcomes with definitions and rationales

Outcome	Definition	Rationale
Effectiveness—outcome measures	What outcomes were used to measure programme effectiveness^a^?	To identify range of outcomes used to measure effectiveness of available interventions
Effectiveness—outcome results	What were the programme effectiveness results?	To evaluate effectiveness of available interventions
Acceptability—outcome measures	What outcomes were used to measure programme acceptability^b^?	To identify range of outcomes used to measure acceptability of available interventions
Acceptability—outcome results	What were the programme acceptability results?	To evaluate acceptability of available interventions
Feasibility—outcome measures	What outcomes were used to measure programme feasibility^c^?	To identify range of outcomes used to measure feasibility of available interventions
Feasibility—outcome results	What were the programme feasibility results?	To evaluate feasibility of available interventions

^a^The degree to which the programme has reduced and prevented mental ill-health and substance use among participants

^b^The degree to which programme users are satisfied

^c^The degree to which the logistical delivery of the programme was achievable and sustainable, with consideration of required land, resources, and capital. This includes the level of attendance or participation from programme start to completion

### Risk of bias in individual studies

The risk of bias in randomised trials will be assessed using version 2 of the Cochrane risk-of-bias tool for randomised trials (RoB 2), the recommended tool for use in Cochrane reviews. Through systematically identifying features of trial design, process, and results, the RoB 2 tool will generate a judgement about the risk of bias arising from the trial: ‘low’, ‘high’, or ‘some concerns’. The risk of bias in non-randomised studies will be assessed using The Risk of Bias in Non-randomised Studies of Interventions (ROBINS-I) tool as per Cochrane Reviews’ recommendation. The ROBINS-I tool will facilitate domain-targeted judgements specific to non-randomised study design to generate an overall risk of bias judgement: either ‘low’, ‘moderate’, serious’, or ‘critical’. The Joanna Briggs Institute Critical Appraisal Checklist for Quasi-Experimental Studies and The Joanna Briggs Institute Critical Appraisal Checklist for Qualitative Research (CASP) will be used to appraise pre-post design (quasi-experimental) studies and qualitative studies, respectively, generating a recommendation to ‘include’, ‘exclude’, or ‘seek further information’. One reviewer conducted risk of bias assessments and a second reviewer verified a random selection of 20% equivalent of included articles.

## Analysis and synthesis

The characteristics of existing trauma-informed mental ill-health and/or substance use prevention programmes for young people will be summarised in accordance with the standardised data extraction form outlined in Tables 3 and 4. Proportions of characteristics among programmes will be quantitatively described. Observable similarities and differences between these characteristics across programmes will also be quantitatively described. Where available, effect sizes will be extracted from studies regarding programme effectiveness, acceptability, and feasibility. Studies reporting will be inspected and all effect estimates pertaining to effect sizes detailed within included studies will be extracted and narratively summarised, including risk factors, odds ratios, and mean differences. Qualitative synthesis will describe and summarise levels of effectiveness, acceptability, and feasibility, and how these factors were measured within each study. The process of qualitative synthesis will seek to describe marked similarities across interventions with positive intervention effects and contextualise these commonalities within the available literature regarding trauma-informed prevention. Through highlighting aspects of intervention effectiveness, these qualitative analyses will underscore future directions for research and intervention work to advance the field of trauma-informed prevention for depression, anxiety, and substance use among young people.

A high level of heterogeneity is anticipated; henceforth, meta-analysis is unlikely. Where possible, the authors will conduct a meta-analysis and synthesise studies within each level of prevention, namely primary prevention effects pooled, secondary prevention effects pooled, and tertiary preventions pooled. Notwithstanding this, qualitative synthesis as described above will circumvent any heterogeneity and capture study details relevant to the research questions.

All members of the project team will contribute to and supervise all aspects of data analysis and synthesis.

## Discussion

This review will advance our understanding of the trauma-informed design principles critical for the development and/or adaptation of effective, acceptable, and feasible trauma-informed mental ill-health and/or substance use prevention programmes. These results will have important equity implications given many young people exposed to early trauma are part of underserved communities that are also at increased risk of mental health and substance use during adolescence, including First Nations communities and sexuality and gender-diverse (LGBTQ +) communities [38, 50, 51]. By organising and summarising the evidence regarding existing effective trauma-informed mental ill-health prevention and substance use for young people, researchers will be better placed to develop, refine, evaluate, and implement interventions designed to interrupt and prevent already elevated trajectories of mental ill-health, substance use, and related harms among those young people exposed to adversity.

## Data Availability

The datasets used and/or analysed during the current study are available from the corresponding author upon reasonable request.
